# Unmasking Individual and Institutional HIV Stigma in Hospitals: Perspectives of Dutch Healthcare Providers

**DOI:** 10.1007/s10461-024-04404-0

**Published:** 2024-06-13

**Authors:** C. C. E. Jordans, K. J. Vliegenthart-Jongbloed, A. W. van Bruggen, N. van Holten, J. E. A. van Beek, M. Vriesde, D. van der Sluis, A. Verbon, A. H. E. Roukens, S. E. Stutterheim, C. Rokx

**Affiliations:** 1 Department of Medical Microbiology and Infectious Diseases, Erasmus University Medical Center, P.O. Box 2040, Rotterdam, 3015 CN The Netherlands; 2 Department of Internal Medicine, Section Infectious Diseases, Erasmus University Medical Center, Rotterdam, 3015 CN The Netherlands; 3 Master student Infectious Diseases, Erasmus University Medical Center, Rotterdam, The Netherlands; 4 Department of Infectious Diseases, Leiden University Medical Center, Leiden, The Netherlands; 5 Department of Infectious Diseases, University Medical Center Utrecht, Utrecht, The Netherlands; 6 Department of Health Promotion & Care and Public Health Institute, Maastricht University, PO Box 616, Maastricht, 6200 MD The Netherlands

**Keywords:** HIV stigma, Healthcare, Healthcare providers, HIV

## Abstract

People with HIV continue to experience HIV stigma. Quantitative data on HIV stigma perpetrated by healthcare providers of hospitals providing HIV care in high-income countries are limited. The aim of this study is to investigate factors associated with HIV stigma in Dutch healthcare settings from the healthcare providers’ perspective. We conducted a cross-sectional study using the questionnaire ‘Measuring HIV Stigma and Discrimination Among Health Facility Staff – Monitoring Tool for Global Indicators’ to assess HIV stigma among healthcare providers (n = 405) in two academic hospitals. Healthcare providers licensed to provide medical care were eligible for inclusion. The primary outcome was the self-reported prevalence of at least one manifestation of HIV stigma measured by six stigma indicators (four individual, two institutional). Secondary outcomes were the prevalence of HIV stigma per indicator, per occupation, per department, and factors associated with individual stigma indicators. HIV stigma was prevalent among 88.1% (95%CI 84.5% − 91.2%) of participants. Stigma was mostly driven by negative attitudes towards people with HIV and worry to acquire HIV. Multivariate analysis showed that several factors were associated with HIV stigma, including younger age, male sex, working at one of the surgical departments, and working as a nurse. Having received any training on HIV stigma and/or discrimination was associated with less HIV stigma among all indicators. In conclusion, HIV stigma is highly prevalent among Dutch healthcare providers. Targeted approaches, including training on HIV stigma and discrimination, are needed to reduce HIV stigma in healthcare and should, among others, focus on younger healthcare providers.

**Supplementary Information:**

The online version contains supplementary material available at 10.1007/s10461-024-04404-0.

## Background

Over the last three decades, the human immunodeficiency virus (HIV) epidemic has emerged as one of the most challenging public health concerns globally [1]. Therefore, the Joint United Nations Programme on HIV/acquired immunodeficiency syndrome (AIDS) (UNAIDS) has set the 95-95-95 goals to end AIDS by 2030. These cascade of care goals mean that by the end of 2025 at least 95% of all people with HIV should be diagnosed, of which 95% should be treated with antiretroviral treatment (ART), with 95% having an undetectable plasma viral load [2]. By the end of 2022, the worldwide figure was 86-89-93 [3]. To reach 95% in each pillar, UNAIDS has included a focus in its strategy to address the reduction of inequalities in testing and treatment access within specific subpopulations. This strategy includes the 10-10-10 targets indicating that, by the end of 2025, less than 10% of people with HIV experience stigma and discrimination, less than 10% of the countries criminalize HIV, and less than 10% of people with HIV experience gender-based inequality and violence [4]. However, people with HIV still experience disproportionate intersecting forms of discrimination and stigma based on aspects of their identity, such as sexual orientation, race/ethnicity, gender identity, substance use, or engagement in sex work [5, 6].

Stigma is defined by the Health Policy Project as a social process of devaluing persons, beginning with marking or labelling someone’s differences, then attributing negative connotations or values to those differences. This process leads to distancing and separation of the person, cumulating in discrimination [7]. HIV-related stigma and discrimination are widespread and continue to undermine interventions across the cascade of care, especially in the first two pillars [3, 8, 9]. Additionally, it impacts linkage to HIV prevention and the quality of life of those with and most affected by HIV [10]. Although healthcare providers are expected to provide comfort, support, and encouragement, previous research suggested that they also may engage in stigmatization of people with HIV, fueled by both individual- and institutional-level factors [11]. Stigmatizing behaviors negatively impacted relationships between patients and healthcare providers and were associated with reduced retention in care, underutilization of HIV care services, and suboptimal adherence to ART [12]. Thus, stigma and discrimination among healthcare providers represent a critical barrier to achieving the envisioned care continuum goals and eliminating HIV [9].

The UNAIDS focus on addressing inequalities and stigma is also relevant in the Netherlands. With a current figure of 94-96-96 on the HIV cascade of care goals, the Netherlands is on the right path to reach the triple 95 goals [13]. However, progress has to be made in the first pillar as HIV testing is often not done. This has various reasons, including HIV stigma [9]. Experiences of stigma and discrimination in healthcare from the perspective of people with HIV have been well documented. A recent large international study spanning 25 countries found that 13.0% of people with HIV have recently experienced stigma and discrimination from their healthcare providers associated with their HIV status [14]. This experienced stigma increased considerably when seeking care for non-HIV-related health problems [15, 16]. Stigma and discrimination mostly manifested as verbal abuse, gossip, denial of services, and disclosing a patients’ HIV status without consent [14, 15]. A recent Dutch study on the perspectives of people with HIV showed an increase in HIV stigma in Dutch healthcare settings over the past decade [15]. However, quantitative data on HIV stigma among healthcare providers working in hospitals where people with HIV usually receive their care in high-income countries are nearly absent [17–20]. Most of the studies available were conducted among healthcare providers who worked in hospitals in low- and middle-income countries [21–27]. A knowledge gap remains as to the extent of stigmatic behavior and drivers among hospital staff which hinders developing targeted stigma reduction interventions.

In this study, we investigated manifestations associated with HIV stigma in Dutch healthcare settings from the perspective of healthcare providers to inform on its existence at the individual and institutional level.

## Methods

### Study Design

Between April 2023 and December 2023, we conducted a digital cross-sectional quantitative questionnaire at Erasmus University Medical Center (EMC), Rotterdam and Leiden University Medical Center (LUMC), Leiden in the Netherlands. Our questionnaire was based on the short version of the standardized brief questionnaire for HIV stigma based on the framework for action of the Health Policy Project ‘Measuring HIV Stigma and Discrimination Among Health Facility Staff – Monitoring Tool for Global Indicators’ [28]. We added a section on barriers related to discussing HIV, risk factors, and HIV testing with patients for healthcare providers to give insight on the impact of stigma on HIV testing practices. To ensure relevance and representativeness, the questionnaire was reviewed by a blinded panel consisting of six European HIV medical specialists and an academic HIV stigma expert. This panel scored all questions on the degree of relevance (not relevant to highly relevant) and degree of clarity (not clear to completely clear). All items showed satisfactory scale and item content validity indices (content validity index ≥ 0.83) [29]. Before start of our study, the standardized brief questionnaire of the Health Policy Project had been administered to 60 healthcare providers at EMC and LUMC. We then piloted these results to showcase satisfactory inter-item reliability using Cronbach’s alpha (Table 1).

### Participants

Healthcare providers working in the participating hospitals who were licensed to provide medical care were eligible for inclusion. Participant were categorized as medical doctors, residents, nurses, or other healthcare providers (e.g. midwives, pharmacists, interns).

### Procedures

The link to the digital questionnaire (Limesurvey) was distributed via email by the HIV team to the head of the departments directed at all employees who fit the inclusion criteria as we did not have access to email addresses of all employers because of general data protection regulations. This link was also published on the secured institutional website. To increase the response rate, the questionnaires were distributed during on-site visits, including a short presentation on the #aware.hiv project (www.awarehiv.com), at large departments in the hospitals (cardiology, dermatology, emergency medicine, gastroenterology, infectious diseases, internal medicine, neurosurgery, otorhinolaryngology, and pulmonology).

### Data Collection

The questionnaire covered five main HIV stigma indicators (Table 1, Appendix A) and was supplemented with one section to assess possible barriers to discuss HIV and testing with patients. Baseline information was collected on the following demographic and work characteristics: age, sex, current occupation, department, years worked in healthcare, number of people with HIV directly treated or cared for in the last 12 months, previous training received in HIV stigma and discrimination, infection control, patients’ informed consent, privacy, and confidentiality, and key population stigma and discrimination. Furthermore, data was collected on the main stigma indicators and barriers to discussing HIV. Data were collected in an electronic case record form.

**Table 1:** The six main HIV stigma indicators including inter-item reliability

The HIV stigma indicators measured by an adapted version of the validated questionnaire ‘Measuring HIV stigma and discrimination among health facility staff: indicator monitoring tool’ *				
	Stigma indicator	Measurement	Scale	Cronbach’s alpha
Immediately actionable causes	Fear of HIV infection	Level of worry to acquire HIV when caring for or providing services to people with HIV	4-point Likert Scale: ‘*not worried’*, ‘*a little bit worried*’, ‘*worried*’, and ‘*very worried*’	0.82
	Institutional-level facilitators and barriers (facility policies)	Level of agreement with statements on the extent to which facilities and protocols for HIV infection mitigation are in place	4-point Likert Scale: ‘*strongly agree*’, ‘*agree*’, ‘*disagree*’, and ‘*strongly disagree*’	0.89
		Presence of policies that prevent stigma-related discrimination towards people with HIV	Categorical questions: ‘*yes*’, ’*no*’, and *’don’t know*’	- **
	Attitudes (stereotypes and prejudice)	Level of agreement with stereotypes about, and prejudicial attitudes towards people with HIV	4-point Likert Scale: ‘*strongly agree*’, ‘*agree*’, ‘*disagree*’, and ‘*strongly disagree*’	0.59
Manifestations (discrimination)	Self-reported use of unnecessary infection control measures	Use of unnecessary precautionary measures when providing services to people with HIV	Categorical questions: ‘*yes*’, ’*no*’, and ‘*not applicable*’	- **
	Observed stigma	Observed stigma and/or discrimination in the provision of care for people with HIV by other healthcare providers	4-point Likert Scale: ‘*never*’, ‘*once or twice*’, ‘*several times*’, and ‘*most of the time*’	0.95
Experienced barriers	Barriers discussing HIV and risk factors with patients	Level of difficulty discussing HIV and HIV testing with patients	4-point Likert Scale: ‘*very easy*’, ‘*easy*’, ‘*difficult*’, and ‘*very difficult*’	0.83***

HIV = human immunodeficiency virus

* Reference: https://www.healthpolicyproject.com/pubs/49_StandardizedBriefQuestionnaireMeasuringSD.pdf

** No Cronbach’s alpha was calculated for dichotomous variables

*** As this question was not included in the pilot, we used the answers of the first 60 participants to calculate this Cronbach’s alpha

### Outcomes

The primary outcome was the prevalence of participants reporting at least one sign of HIV stigma measured by the following stigma indicators: fear of HIV infection, attitudes towards people with HIV, use of unnecessary infection control measures, and barriers discussing HIV and risk factors with patients. Secondary outcomes were the difference in the prevalence of HIV stigma per stigma indicator, by occupation, and by department. Outcome variables were defined as having any worry to acquire HIV, any supplies/protocols available to reduce the risk of acquiring HIV, any policies available on discrimination of people with HIV, any negative attitudes towards people with HIV, any use of unnecessary precautionary measures, any observed stigma and discrimination, and any difficulties discussing HIV and HIV-related matters with patients.

### Statistical Analysis

Descriptive statistics are characterized as number (%) for categorical variables and median and interquartile range (IQR) for continuous variables. We dichotomized outcomes of the Likert Scale in two ordinal categories: ‘worried’ and ‘not worried’ for *fear of HIV infection*, ‘agree’ and ‘disagree’ for *institutional-level facilitators and barriers* and *attitudes towards people with HIV*, ‘never’ and ‘at least once’ for *observed and anticipated stigma*, and ‘easy’ and ‘ difficult’ for *barriers discussing HIV with patients*. Answer options ‘not applicable’ or ‘I do not provide care to patients with HIV’ for the indicators fear of HIV infection, infection precaution measures, and experienced barriers discussing HIV and HIV risk factors were excluded for analysis as per Health Policy Project guidance. Answer option ‘don’t know’ for the indicators policies available to prevent stigma against people with HIV and experienced barriers discussing HIV and HIV risk factors were included for analysis as recommended by the user manual. Missing data was included in the analysis as recommended by the Health Policy Project guidance [7]. Participants who completed at least one of the questions on the demographics were included in the baseline characteristics. Participants who completed at least one of the stigma questions were included for outcome analysis. The primary outcome was constructed out of the following variables: any worry to acquire HIV, any negative attitudes towards people with HIV, any use of unnecessary precautionary measures, and any difficulties discussing HIV and risk factors. Chi-square tests were used for univariate analyses per stigma indicator. A multivariate logistic regression analysis was used to study factors associated with HIV stigma indicators reflecting the behavior or beliefs of the healthcare providers (fear of HIV infection, attitudes (stereotypes and prejudice), self-reported use of unnecessary infection control measures, and barriers discussing HIV and risk factors with patients). In the multivariate analysis departments were grouped in three groups: surgical departments (cardiothoracic surgery, gynecology, neurosurgery, ophthalmology, otorhinolaryngology, and surgery), acute departments (emergency medicine, intensive care unit, and short stay), and medical departments (cardiology, dermatology, endocrinology, gastroenterology, geriatrics, hematology, infectious diseases, internal medicine, medical microbiology, nephrology, neurology, oncology, pediatrics, psychiatrics, and pulmonology). Occupation was grouped in four groups: medical specialist, resident (resident in training and resident not in training), nurse (physician assistant, specialized nurse, and nurse), and other (medical intern, medical researcher, nurse intern, and researcher).

A p-value of ≤ 0.05 was considered statistically significant. Data from LimeSurvey were tabulated and aggregated in a Microsoft Excel spreadsheet (Microsoft, Redmond, WA, USA). Statistical analyses were conducted using SPSS version 28 (IBM, Armonk, NY, USA).

### Ethical Considerations

The study procedures were carried out in accordance with the regulations of the Declaration of Helsinki. Approval of the research Medical Ethics Committee of the faculty of medicine, EMC, Rotterdam, was obtained (MEC-2020-0140). The Medical Ethics Committee determined that this research did not meet the definition of medical research involving human subjects under Dutch law (WMO). Informed consent was obtained from all participants before data collection.

## Results

### Baseline Characteristics

The questionnaire received 450 responses. Of these, 45 (10.0%) were excluded due to failure to complete any of the questions. A total of 405 participants were included of whom the baseline characteristics are shown in Table 2. Most of the participants were female (72.8%) and worked as a nurse (49.9%). The median age of the participants was 33 years (IQR 28–40). Participants have worked in healthcare for a median of 9 years (IQR 5–17) and treated median 3 patients with HIV per year (IQR 1–6). Notably, 13.9% of participants had not provided care to a person with HIV in the past year while 4.5% of participants cared for at least 50 people with HIV in the past year. Most participants worked at the internal medicine department (19.0%), the emergency department (8.6%), and the cardiothoracic surgery department (8.4%). Overall, around one in ten participants were deployed, either partially or entirely, within the infectious diseases department. Of all participants, 10.6% had, at some point in time, received training specifically addressing HIV stigma and discrimination and 41.5% had received training on infection control and universal precautions.

**Table 2:** Baseline characteristics of participants

	Participants
*Age, years*	
Median (IQR)	33 (28–40)
*Age groups, years, n (%)*	
≤ 25	68 (16.8)
26–45	268 (66.2)
≥ 46	69 (17.0)
*Gender, n (%)*	
Female	295 (72.8)
Male	108 (26.7)
Other	2 (0.5)
*Occupation, n (%)*	
Nurse	202 (49.9)
Resident	96 (23.7)
Medical specialist	67 (16.5)
Other*	40 (9.9)
*Department(s), n (%)*	
Internal medicine	77 (19.0)
Emergency department	35 (8.6)
Cardiothoracic surgery	34 (8.4)
Pulmonology	32 (7.9)
Otorhinolaryngology	28 (6.9)
Oncology	25 (6.2)
Surgery	24 (5.9)
Gastroenterology/hepatology	24 (5.9)
Neurosurgery	23 (5.7)
Infectious diseases**	20 (4.9)
Intensive care	17 (4.2)
Short stay	12 (3.0)
Dermatology	10 (2.5)
Other***	44 (10.9)
*Affiliated to > 1 department, n (%)*	
Yes	90 (22.2)
*Time worked in healthcare, years*	
Median (IQR)	9 (5–17)
*Time worked in healthcare, years grouped, n (%)*	
< 5	126 (31.1)
5–10	116 (28.7)
> 10	160 (39.5)
Missing	3 (0.7)
*Number of treated patients with HIV per year*	
Median (IQR)	3 (1–6)
*City of work, n (%)*	
Rotterdam	308 (76.0)
Leiden	96 (23.7)
Missing	1 (0.3)
*Received training on the following topics*	
HIV stigma and discrimination	43 (10.6)
Infection control and universal precautions	168 (41.5)
Patient’s informed consent, privacy, and confidentiality	154 (38.0)
Key population stigma and discrimination	57 (14.1)
Received any of above training	223 (55.1)

HIV = human immunodeficiency virus

* Other occupation included: medical intern, medical researcher, nurse intern, researcher

** These healthcare providers work solely at the infectious diseases department

*** Other included: cardiology, endocrinology, geriatrics, gynecology, hematology, medical microbiology, nephrology, neurology, ophthalmology, pediatrics, psychiatrics, and other

### HIV Stigma

Overall, 88.1% (95% confidence interval (CI) 84.5 − 91.2%) of the participants reported any manifestation of HIV stigma. Furthermore, HIV stigma was prevalent among all investigated stigma indicators (Table 3) and their associations with baseline characteristics in the multivariate analysis are provided in Table 4. In total 388 (95.8%) participants completed at least one of the stigma-related questions. The 17 participants who did not complete any of the stigma-related questions were comparable on all characteristics except having more often an ‘other’ occupation (supplementary Table 1).

**Table 3:** Prevalence of HIV stigma indicators

The HIV stigma indicators measured by an adapted version of the validated questionnaire ‘Measuring HIV stigma and discrimination among health facility staff: indicator monitoring tool’ *					
*Immediately actionable causes*					
*Fear of HIV infection*					
Level of worry when conducting the following activities:	Not worried, n (%)	Worried, n (%)	Not applicable, n **		
Touch clothing of a patient with HIV	350 (90.4)	37 (9.6)	1		
Dress wounds of a patient with HIV	127 (34.6)	240 (65.4)	21		
Draw blood from a patient with HIV	101 (27.4)	267 (72.6)	20		
**Any worry to acquire HIV**	**No, n (%)** **110 (28.4)**	**Yes, n (%)** **277 (71.6)**	**Not** applicable, n ** **1**		
*Institutional-level facilitators and barriers (facility policy)*					
Level of agreement with the following statements:	Agree, n (%)	Disagree, n (%)	Missing, n (%)		
There are adequate supplies in my health facility that reduce my risk of acquiring HIV	361 (93.0)	18 (4.7)	9 (2.3)		
There are standardized procedures/protocol in my health facility that reduce my risk of acquiring HIV	353 (91.0)	26 (6.7)	9 (2.3)		
**Any supplies/protocols to reduce risk to acquire HIV**	**Yes, n (%)** **370 (95.4)**	**No, n (%)** **9 (2.3)**	**Missing, n (%)** **9 (2.3)**		
Health facility’s policies on discrimination	No, n (%)	Yes, n (%)	Don’t know, n (%)		Missing, n (%)
I will get in trouble at work if I discriminate against patients with HIV	16 (4.1)	217 (55.9)	145 (37.4)		10 (2.6)
My health facility has written guidelines to protect patients with HIV from discrimination	14 (3.6)	68 (17.5)	297 (76.6)		9 (2.3)
**Any policies on discrimination**	**No, n (%)** **14 (3.6**)	**Yes, n (%)** **242 (62.4)**	**Don’t know, n (%)** **124 (32.0)**		**Missing,** **n (%)** **8 (2.0)**
*Attitudes (stereotype and prejudice*					
Level of agreement with the following statements:	Agree, n (%)	Disagree, n (%)	Missing, n (%)		
Most people with HIV do not care if they infect other people	27 (6.9)	344 (88.7)	17 (4.4)		
People with HIV should feel ashamed of themselves	3 (0.8)	368 (94.8)	17 (4.4)		
People acquire HIV because they engage in irresponsible behaviors	38 (9.8)	332 (85.6)	18 (4.6)		
Women with HIV should be allowed to have babies if they wish**	329 (84.8)	41 (10.6)	18 (4.6)		
**At least one negative attitud** **e**	**Yes, n (%)** **87 (22.4)**	**No, (%)** **284 (73.2)**	**Missing, n (%)** **17 (4.4)**		
*Manifestations (discrimination)*					
*Self-reported use of unnecessary infection control measures*					
Typically use any of the following measures when providing services to patients with HIV:	No, n (%)	Yes, n (%)	Not applicable, n **		
Avoid physical contact	347 (93.5)	24 (6.5)	17		
Wear double gloves	312 (84.6)	57 (15.4)	19		
**Any use of unnecessary precautionary measures**	**No, n (%)** **297 (80.1)**	**Yes, n (%)** **74 (19.9)**	Not applicable, n ** **17**		
*Observed discrimination*					
In < 12 months have you seen a person with HIV	Yes, n (%)	No, n (%)	Don’t know, n (%)		
Have you seen a person with HIV in your health facility	303 (78.1)	56 (14.4)	29 (7.5)		
In < 12 months how often observed the following at your health facility	Never, n (%)	At least once, n (%)	Missing, n (%)		
Healthcare providers unwilling to care for a patient with HIV	290 (74.7)	12 (3.1)	86 (22.2)		
Healthcare providers providing poorer quality of care to a patient with HIV compared to other patients	279 (71.9)	24 (6.2)	85 (21.9)		
**Any observed stigma and discrimination**	**No, n (%)** **274 (70.6)**	**Yes, n (%)** **29 (7.5)**	**Missing, n (%)** **85 (21.9)**		
*Experienced barriers*					
*Discussing HIV and risk factors with patients*					
Level of difficulty for healthcare providers discussing the following topics:	Easy, n (%)	Difficult, n (%)	Missing, n (%)	Not applicable, n **	
The possibility of an HIV infection	168 (53.7)	122 (39.0)	23 (7.3)	75	
The need to test for HIV	243 (76.2)	53 (16.6)	23 (7.2)	69	
Risk factors for HIV	208 (64.8)	90 (28.0)	23 (7.2)	67	
HIV-related topics when a patient is accompanied by a family member	96 (29.6)	205 (63.3)	23 (7.1)	64	
**Any difficulties discussing HIV and risk factors**	**No, n (%)** **86 (25.7)**	**Yes, n (%)** **225 (67.4)**	**Missing,** **n (%)** **23 (6.9)**	**Not applicable,** n ** **54**	

HIV = human immunodeficiency virus

* Reference: https://www.healthpolicyproject.com/pubs/49_StandardizedBriefQuestionnaireMeasuringSD.pdf

** Not applicable will be excluded from the denominator, as the response to this question does not apply to the healthcare provider answering this question

*** This statement was inverted in the ‘any negative attitudes’ section as women with HIV should be allowed to have babies is a positive attitude towards people with HIV

### Immediate Actionable Causes of HIV Stigma and Discrimination

#### Fear of HIV Infection

A majority (71.6%) of healthcare providers were worried to acquire HIV when caring for a patient with HIV. This was driven by fear when drawing blood (72.6%) and dressing wounds (65.4%). Of all participants, 46 healthcare providers (12.5%) reported being very worried to acquire HIV when drawing blood from patients with HIV and 31 (8.4%) were very worried to acquire HIV when dressing wounds of patients with HIV (supplementary Table 2). Almost all healthcare providers ≤ 25 years expressed worries to acquire HIV (96.9%) and participants from this age group worried considerably more than those 46 years and older (adjusted odds ratio (aOR) = 28.0, 95% CI 5.2–151.0, *p* < 0.01). Healthcare providers from surgical departments worried more about acquiring HIV (aOR = 1.9, 95% CI 1.0–3.8, *p* = 0.05) while training on HIV stigma and/or discrimination and more work experience decreased this worry.

#### Institutional-Level Facilitators and Barriers (Facility Policies)

Almost all participants confirmed adequate supplies (93.0%) and protocols were in place (91.0%) for infection prevention. Around four in ten healthcare providers were either unsure or certain that they would not face reprimands when they discriminate people with HIV. The majority (76.6%) were unaware of any institutional guidelines on discrimination. Awareness on possible consequences of discrimination was more often observed among those that received any form of training on HIV stigma or discrimination (73.2% versus 53.9%, χ2 = 8.18, *p* = 0.04).

#### Attitudes (Stereotypes and Prejudice)

In total 87 (22.4%) healthcare providers showed at least one of the measured negative attitudes towards people with HIV. Most reported negative attitudes were disagreement that women with HIV could have babies (10.6%), belief that people acquire HIV through perceived irresponsible behavior (9.8%), and belief that people with HIV do not care about infecting others (7.0%). Negative attitudes against people with HIV were more common among males (aOR = 2.6, 95% CI 1.5–4.8, *p* < 0.01), and nurses (aOR = 2.8, 95% CI 1.1–6.8, *p* = 0.03) and other professions, including medical interns, medical researchers, nurse interns, and researchers, (aOR = 5.0, 95% CI 1.5–16.9, *p* = 0.01) both compared to medical specialists. Training on HIV stigma and/or discrimination was associated with less negative attitudes (aOR = 0.28, 95% CI 0.09–0.83, *p* = 0.02). We found no clear pattern with age or general work experience in relation to the expression of negative attitudes towards people with HIV.

### Manifestations (Discrimination)

#### Self-Reported Use of Unnecessary Infection Control Measures

Nearly 20% of healthcare providers used any form of unnecessary infection control when caring for people with HIV by using double gloves (15.4%) and/or completely avoiding physical contact (6.5%). This behavior was more prominent in healthcare providers from surgical departments (cardiothoracic surgery, gynecology, neurosurgery, ophthalmology, otorhinolaryngology, and surgery) (aOR = 2.9, 95% CI 1.6–5.4, *p* < 0.01) compared to medical departments (cardiology, dermatology, endocrinology, gastroenterology, geriatrics, hematology, infectious diseases, internal medicine, medical microbiology, nephrology, neurology, oncology, pediatrics, psychiatrics, and pulmonology). Additionally, younger healthcare providers (≤ 25 years) tended to use more unnecessary precautionary measures (aOR = 3.1, 95% CI 0.9–11.3, *p* = 0.08). Furthermore, healthcare providers without training on HIV stigma and/or discrimination reported more unnecessary precautionary measures (aOR = 5.2, 95% CI 1.1–23.6, *p* = 0.03).

#### Observed Stigma

Nearly 1 in 10 healthcare providers (7.5%) reported having observed any stigma and discrimination in their health facility over the last 12 months. Most observed were healthcare providers providing poorer quality of care to people with HIV compared to other patients (6.2%). Those who had observed stigma more often worked at the infectious diseases department (20.0% versus 8.0%, χ2 = 5.8, *p* = 0.02), provided care for more people with HIV (12.5% versus 8.4%, χ2 = 1.23, *p* = 0.268), and had received training on this topic (23.5% versus 7.8%, χ2 = 8.62, *p* < 0.01). 

### Experienced Barriers Discussing HIV and Risk Factors

#### Barriers Discussing HIV and Risk Factors with Patients

Most healthcare providers (67.4%) expressed some degree of difficulty in discussing HIV, testing, or HIV-related matters with patients. Approximately one-third of the healthcare providers (39.0%) found it difficult to discuss the possibility of an HIV infection with their patients and 28.0% had difficulties discussing risk factors for HIV. In contrast, discussing the need to test for HIV was found relatively easy (76.2%). The most common barrier was discussing HIV-related topics when a patient was accompanied by a family member (63.3%), with 10.5% reporting this aspect as very difficult. Having received training on HIV stigma and/or discrimination was the only identified factor that decreased these barriers to discuss HIV-related matters (aOR = 2.5, 95% CI 1.1–5.5, *p* = 0.02).

**Table 4:** Factors associated with HIV stigma indicators

	Fear of HIV infection			Attitudes (stereotypes and prejudice)			Self-reported use of unnecessary infection control measures			Experienced barriers		
Characteristic	**Any worry to acquire HIV**			**At least one negative attitude towards people with HIV**			**Any taken unnecessary precautionary measures**			**Any difficulties discussing HIV and risk factors**		
Number of events/sample size	277/387			87/371			74/371			225/311		
	aOR	95% CI	*p*-value	aOR	95% CI	*p*-value	aOR	95% CI	*p*-value	aOR	95% CI	*p*-value
*Age (years)*												
≤ 25	28.0	5.2–151.0	< 0.01*	1.9	0.6–6.1	0.29	3.1	0.9–11.3	0.08	0.9	0.3–3.1	0.87
26–45	5.3	2.6–10.7	< 0.01*	1.1	0.5–2.7	0.80	1.5	0.6–3.8	0.45	1.1	0.5–2.4	0.90
≥ 46 (ref.)												
*Gender*												
Male	0.8	0.4–1.4	0.46	2.6	1.5–4.8	< 0.01*	1.3	0.7–2.6	0.37	0.7	0.4–1.2	0.19
Female (ref.)												
*Occupation*												
Nurse	1.0	0.5–2.1	0.90	2.8	1.1–6.8	0.03*	0.8	0.3–1.9	0.57	1.5	0.7–3.3	0.28
Resident	0.8	0.3–1.8	0.55	2.2	0.8–6.2	0.15	0.7	0.2–1.9	0.42	1.0	0.4 − 2.5	0.98
Other**	0.7	0.2–2.5	0.56	5.0	1.5–16.9	0.01*	0.8	0.2–3.0	0.77	1.3	0.4–4.7	0.65
Medical specialist (ref.)												
Departments***												
Surgical	1.9	1.0–3.8	0.05*	0.7	0.4–1.3	0.27	2.9	1.6–5.4	< 0.01*	1.0	0.5–2.0	0.89
Acute	1.6	0.7–3.4	0.23	1.4	0.6–3.0	0.41	0.5	0.2–1.5	0.24	0.9	0.5–1.9	0.75
Medical (ref.)												
*Infectious diseases department*												
No	1.0	0.4–2.6	0.95	0.5	0.2–1.2	0.11	1.7	0.5–6.5	0.41	1.5	0.7–3.6	0.33
Yes (ref.)												
*Years worked in healthcare*												
≤ 5	2.9	1.2–7.4	0.02*	1.1	0.5–2.7	0.80	1.4	0.5–3.6	0.53	1.1	0.4–2.7	0.86
6–10	1.4	0.7–2.8	0.33	1.6	0.8–3.3	0.23	1.1	0.5–2.5	0.84	1.2	0.6–2.7	0.59
≥ 10 (ref.)												
*Number of treated HIV patients in the last 12 months*												
< 6 patients	1.3	0.7–2.4	0.38	1.5	0.8–2.9	0.24	1.1	0.5–2.4	0.78	1.1	0.6–2.1	0.71
≥ 7 patients (ref.)												
*Received training on HIV stigma and/or discrimination*												
No	2.1	0.9–5.1	0.09	3.6	1.2–11.3	0.02*	5.2	1.1–23.6	0.03*	2.5	1.1–5.5	0.02*
Yes (ref.)												

HIV = human immunodeficiency virus

* Significant (*p* ≤ 0.05)

** Other occupation included: medical intern, medical researcher, nurse intern, researcher

*** **Surgical** departments included: cardiothoracic surgery, gynecology, neurosurgery, ophthalmology, otorhinolaryngology, and surgery, **acute** departments included: emergency medicine, intensive care unit, and short stay, **medical** departments included: cardiology, dermatology, endocrinology, gastroenterology, geriatrics, hematology, infectious diseases, internal medicine, medical microbiology, nephrology, neurology, oncology, pediatrics, psychiatrics, and pulmonology

## Discussion

Our study showed that almost all healthcare providers reported any manifestation of HIV stigma, either at individual level or at institutional level. The most prevalent drivers of stigma at individual level were reflected by negative attitudes towards people with HIV and worry to acquire HIV. Furthermore, enacted stigma by discriminative or unnecessary cautious behavior was frequently disclosed. Stigma was most prominently driven by younger healthcare providers, men, and nurses. Reassuringly, training on HIV stigma and/or discrimination consistently reduced levels of HIV stigma among all measured stigma indicators in healthcare providers.

HIV stigma was more prevalent among certain subgroups. First, stigmatic thoughts and behavior were more frequently disclosed by younger healthcare providers. These findings contrasted to other studies pinpointing stigmatization more among older healthcare providers [18, 19]. Reasons for this difference might be less work experience, limited exposure to people with HIV, and limited knowledge on HIV transmission and HIV stigma. The increased stigma among the future generation of healthcare providers is, however, a relevant signal. We speculate whether this is caused by decreasing levels of HIV knowledge in contemporary times or less training in the educational system, which needs further study. The lack of understanding of infection control was more prominently observed in younger participants which supports this hypothesis. Second, nurses and males expressed more stigmatizing attitudes. This is in line with findings from studies conducted in non-European settings [18–21, 25, 27]. The exact reason for this pattern is unclear but it might reflect different levels of education and social background, including men perpetrating more stigma towards gay men [30]. Third, participants from surgical departments were more likely to take unnecessary precautionary measures which was expected based on the limited data available [17, 18]. Work-related aspects, including more frequent exposure to handling blood, might have caused this. Notably, two in three healthcare providers in hospitals reported any discomfort discussing a possible HIV infection or HIV-related risk factors with their patients.

Importantly, our findings stressed the relevance of training on HIV stigma and/or discrimination in all stigma domains and for all participants. The observed protective effect of training on all stigma indicators and identified groups at risk for stigmatic thoughts and behaviors support the continued implementation of training for all healthcare providers as well as developing targeted approaches for those at risk to express stigma. Prior research indicated that education and interventions concerning HIV risk assessment influenced healthcare providers behavior, fostering confidence in assessing the sexual history of high-risk patients. Additionally, such interventions contribute to an enhanced level of comfort discussing HIV prevention and risk factors [31, 32]. Furthermore, research showed that the lack of HIV knowledge, including awareness of the undetectable = untransmittable (U = U) message, partly explained HIV stigma [19]. Therefore future interventions should include the following aspects: awareness of HIV stigma, fear of acquiring HIV, including addressing knowledge gaps about HIV transmission and U = U, tools to be comfortable assessing the sexual history, and awareness of stigmatizing attitudes. Prior research showed that interventions including these aspects were proven to be effective to reduce HIV stigma in non-European settings [33–35].

This study has some strengths and limitations. First, its main strength is that it encompassed a large variety of specialties and healthcare providers in hospitals, unlike the few prior hospital-based studies available that focused on a single department. Our study complements the field with data from a clinical setting in high-income countries, which was lacking. Second, it provides an evidence based on which groups to target for stigma reduction programs and on the positive association of using specific trainings on HIV stigma. Regarding limitations, the generalizability of the findings to other settings might be limited due to the hospital-based settings and the number of participating centers. Second, although our study is based on a validated questionnaire, responses were self-reported and unsupervised by the researchers which could lead to reporting and social desirability biases. We implemented standardized procedures (appendix B) in approaching participants to decrease social desirable answers, for example by avoiding the explicit mention of the word ‘stigma’. Third, no data was collected on social background. As this might be of influence on HIV stigma, we recommend future studies to include this aspect in the baseline demographics. Fourth, the question in the questionnaire on any worry regarding the procedure of drawing blood from a patient with HIV could have been better specified. Most participants answered this question positively, however, this might be different when more clinical circumstances, e.g. detectable or undetectable viral load, were provided. Last, multiple stigma questionnaires exist. However, the questionnaire we used was validated in six different countries and designed to be administered across all levels and types of healthcare staff. Therefore, we considered this questionnaire suitable as it enables a systemic assessment of HIV stigma.

## Conclusion

HIV stigma is highly prevalent among healthcare providers working in hospitals in the Netherlands. Targeted approaches, including training on HIV stigma and discrimination, are needed to reduce HIV stigma in healthcare and should, among others, focus on younger healthcare providers.

## Electronic Supplementary Material

Below is the link to the electronic supplementary material.

Supplementary Material 1

Supplementary Material 2

Supplementary Material 3

Supplementary Material 4

## References

[1] Ortblad KF, Lozano R, Murray CJ. The burden of HIV: insights from the global burden of Disease Study 2010. Aids Aug. 2013;24(13):2003–17. 10.1097/QAD.0b013e328362ba67 PMC3748855 23660576

[2] UNAIDS. *AIDS Targets: 2025 - Target setting and 2020–2030 resource needs and impact estimation*2021.

[3] UNAIDS. *The path that ends AIDS: UNAIDS global AIDS update 2023*2023.

[4] UNAIDS. *Global AIDS Strategy 2021–2026 - End Inequalities. End AIDS* UNAIDS.org2021.

[5] Stangl AL, Earnshaw VA, Logie CH et al. The Health Stigma and Discrimination Framework: a global, crosscutting framework to inform research, intervention development, and policy on health-related stigmas. *BMC Medicine* 2019/02/15 2019;17(1):31. 10.1186/s12916-019-1271-3 PMC6376797 30764826

[6] Turan JM, Elafros MA, Logie CH et al. Challenges and opportunities in examining and addressing intersectional stigma and health. *BMC Medicine* 2019/02/15 2019;17(1):7. 10.1186/s12916-018-1246-9 PMC6376691 30764816

[7] Jain A, Carr D, Nyblade L. *Measuring HIV stigma and discrimination among Health Facility Staff: standardized brief questionnaire user guide*. : Futures Group, Health Policy Project;2015.

[8] Turan B, Budhwani H, Fazeli PL, et al. How does Stigma affect people living with HIV? The mediating roles of internalized and anticipated HIV Stigma in the effects of Perceived Community Stigma on Health and Psychosocial outcomes. AIDS Behav Jan. 2017;21(1):283–91. 10.1007/s10461-016-1451-5 PMC5143223 27272742

[9] Sullivan MC, Rosen AO, Allen A, et al. Falling short of the First 90: HIV Stigma and HIV Testing Research in the 90-90-90 era. AIDS Behav Feb. 2020;24(2):357–62. 10.1007/s10461-019-02771-7 31907675

[10] Greenwood GL, Wilson A, Bansal GP, et al. HIV-Related Stigma Research as a Priority at the National Institutes of Health. AIDS Behav 2022/01/01. 2022;26(1):5–26. 10.1007/s10461-021-03260-6 PMC8060687 33886010

[11] Geter A, Herron AR, Sutton MY. HIV-Related Stigma by Healthcare Providers in the United States: a systematic review. AIDS Patient Care STDS Oct. 2018;32(10):418–24. 10.1089/apc.2018.0114 PMC6410696 30277814

[12] Rueda S, Mitra S, Chen S, et al. Examining the associations between HIV-related stigma and health outcomes in people living with HIV/AIDS: a series of meta-analyses. BMJ Open Jul. 2016;13(7):e011453. 10.1136/bmjopen-2016-011453 PMC4947735 27412106

[13] van Sighem AI, WFWNM, Boyd A, Smit C, Jongen VW, Matser A. *Monitoring Report 2023. Human Immunodeficiency Virus (HIV) Infection in the Netherlands*2023.

[14] (GNP+) GNoPLwH. *People living with HIV Stigma Idex 2.0. Global report 2023. Hear us out: Community Measuring HIV-Related Stigma and Discrimination*2023.

[15] Stutterheim SE, Kuijpers KJR, Waldén MI, Finkenflügel RNN, Brokx PAR, Bos AER. Trends in HIV Stigma Experienced by People Living With HIV in the Netherlands: A Comparison of Cross-Sectional Surveys Over Time. *AIDS Education and Prevention* 2022/02/01 2022;34(1):33–52. 10.1521/aeap.2022.34.1.33 35192394

[16] Crockett KB, Turan B, Whitfield S, et al. Patient and provider perspectives on HIV Stigma in Healthcare settings in Underserved areas of the US South: a mixed methods study. AIDS Behav 2022/01/01. 2022;26(1):112–24. 10.1007/s10461-021-03470-y PMC9009188 34581951

[17] Moseholm E, Wilken-Jensen C, Weis N. HIV-related stigma among healthcare providers working within infectious diseases and gynecology and obstetrics at a large teaching hospital in Denmark. *AIDS Care* 2023/05/04 2023;35(5):705–713. 10.1080/09540121.2022.2121955 36161975

[18] Houston P, Powell E, Khan J et al. Thirty-five years later: HIV Stigma in Washington, DC Health Care Workers. J Assoc Nurses AIDS Care 2019;30(3). 10.1097/JNC.0000000000000060 30768434

[19] Spence AB, Wang C, Michel K, et al. HIV related stigma among Healthcare providers: opportunities for Education and Training. J Int Association Providers AIDS Care (JIAPAC). 2022;21:23259582221114797. 35850610 10.1177/23259582221114797 PMC9310064

[20] Stringer KL, Turan B, McCormick L, et al. HIV-Related Stigma among Healthcare providers in the Deep South. AIDS Behav 2016/01/01. 2016;20(1):115–25. 10.1007/s10461-015-1256-y PMC4718797 26650383

[21] Galal YS, Khairy WA, Mohamed R, et al. HIV-related stigma and discrimination by healthcare workers in Egypt. Trans R Soc Trop Med Hyg. 2022;116(7):636–44. 34999841 10.1093/trstmh/trab188

[22] Zarei N, Joulaei H, Darabi E, Fararouei M. Stigmatized attitude of Healthcare providers: a Barrier for Delivering Health Services to HIV positive patients. Int J Community Based Nurs Midwifery Oct. 2015;3(4):292–300. PMC4591575 26448956

[23] Rana BK, Sarfraz M, Reza TE, Emmanuel F. A cross-sectional study to assess HIV/AIDS-Related Stigma and its drivers among Dental Healthcare providers in Islamabad, Pakistan. Cureus Oct. 2023;15(10):e46769. 10.7759/cureus.46769 PMC10632562 37954825

[24] Hidayat J, Chen MY, Maulina R, Nurbaya S. Factors Associated with HIV-Related Stigma among Indonesian Healthcare workers: a cross-sectional online survey. J Nurs Res Oct. 2023;1(5):e295. 10.1097/jnr.0000000000000573 37668415

[25] Tavakoli F, Karamouzian M, Rafiei-Rad AA, et al. HIV-Related Stigma among Healthcare providers in different Healthcare settings: a cross-sectional study in Kerman, Iran. Int J Health Policy Manag Apr. 2020;1(4):163–9. 10.15171/ijhpm.2019.92 PMC7182146 32331496

[26] Shah S, Elgalib A, Al-Wahaibi A, et al. Knowledge, attitudes and practices related to HIV Stigma and Discrimination among Healthcare Workers in Oman. Sultan Qaboos Univ Med J Feb. 2020;20(1):e29–36. 10.18295/squmj.2020.20.01.005 PMC7065692 32190367

[27] Ekstrand ML, Ramakrishna J, Bharat S, Heylen E. Prevalence and drivers of HIV stigma among health providers in urban India: implications for interventions. J Int AIDS Soc. 2013;16(3S2):18717. 24242265 10.7448/IAS.16.3.18717 PMC3833193

[28] Project HP. Measuring HIV stigma and discrimination among Health Facility Staff: standardized brief questionnaire. Washington, DC: Futures group, Health Policy Project; 2013.

[29] Yusoff MSB. ABC of Content Validation and Content Validity Index calculation. Educ Med J 2019.

[30] Bettinsoli ML, Suppes A, Napier JL. Predictors of attitudes toward Gay men and Lesbian women in 23 countries. Social Psychol Personality Sci. 2020;11(5):697–708. 10.1177/1948550619887785

[31] Dodge WT, BlueSpruce J, Grothaus L et al. Enhancing primary care HIV prevention: A comprehensive clinical intervention. *American Journal of Preventive Medicine* 2001/04/01/ 2001;20(3):177–183. 10.1016/s0749-3797(00)00308-1 11275443

[32] Verrastro V, Saladino V, Petruccelli F, Eleuteri S. Medical and Health Care Professionals’ Sexuality Education: State of the Art and Recommendations. Int J Environ Res Public Health Mar 25 2020;17(7). 10.3390/ijerph17072186 PMC7177861 32218258

[33] Nyblade L, Srinivasan K, Mazur A, et al. HIV Stigma Reduction for Health Facility Staff: development of a blended- learning intervention. Front Public Health. 2018;6:165. 29977887 10.3389/fpubh.2018.00165 PMC6021510

[34] Pollack TM, Duong HT, Nhat Vinh DT, et al. A pretest-posttest design to assess the effectiveness of an intervention to reduce HIV-related stigma and discrimination in healthcare settings in Vietnam. J Int AIDS Soc. 2022;25(S1):e25932. 35818864 10.1002/jia2.25932 PMC9274370

[35] Anna M, Babu Lal B, Courtney B, Megha S. Impact of ‘HIV-related stigma-reduction workshops’ on knowledge and attitude of healthcare providers and students in Central India: a pre-test and post-test intervention study. BMJ Open. 2020;10(4):e033612. 10.1136/bmjopen-2019-033612 PMC7201299 32284388

