# Supplementary material for: Unmasking Individual and Institutional HIV Stigma in Hospitals: Perspectives of Dutch Healthcare Providers
Source: AIDS Behav. 2024 Jun 13;28(9):3184–95. doi: 10.1007/s10461-024-04404-0 (PMC11390866; doi:10.1007/s10461-024-04404-0)
Supplement: Supplementary file 1 — Supplementary Material 1 [file 10461_2024_4404_MOESM1_ESM.docx]

**Unmasking individual and institutional HIV stigma in hospitals: Perspectives of Dutch healthcare providers**

**Short title: HIV stigma among Dutch healthcare providers**

C.C.E. Jordans^1^, K.J. Vliegenthart-Jongbloed^2^, A.W. van Bruggen^3^, N. van Holten^4^, J.E.A. van Beek, M. Vriesde, D. van der Sluis^4^, A. Verbon^1,5^, A.H.E. Roukens^4^, S.E. Stutterheim^6^, C. Rokx^1,2^

On behalf of the #aware.hiv study group

^1^ Department of Medical Microbiology and Infectious Diseases, Erasmus University Medical Center, 3015 CN, Rotterdam, the Netherlands
^2^ Department of Internal Medicine, Section Infectious Diseases, Erasmus University Medical Center, 3015 CN, Rotterdam, the Netherlands
^3^ Master student Infectious Diseases, Erasmus University Medical Center, Rotterdam, the Netherlands
^4^ Department of Infectious Diseases, Leiden University Medical Center, Leiden, the Netherlands

^5^ Department of Infectious Diseases, University Medical Center Utrecht, Utrecht, the Netherlands
^6^ Department of Health Promotion & Care and Public Health Institute, Maastricht University, PO Box 616, 6200 MD Maastricht, the Netherlands

**Corresponding author:** Dr. C. Rokx, c.rokx@erasmusmc.nl P.O. Box 2040, 3015 CN Rotterdam, the Netherlands, internal postal address Na901K

**Appendix A – questionnaire**

Dear healthcare provider,

The #aware.hiv project does research on the working methods of healthcare providers regarding HIV within the hospital. You can be of great value for this research by completing this questionnaire.

The questionnaire will take approximately 5 till 10 minutes of your time. We would like to mention that while answering the questions, it is not possible to navigate back to previous questions. The answer will be processed anonymously. By starting the questionnaire on the next page, you agree on anonymous participation in this study.

We thank you very much for your help.

Drs. Carlijn Jordans, MD, clinical researcher

Also on behalf of,

Dr. Casper Rokx, internist-infectiologist, Erasmus MC

Dr. Sarah Stutterheim, associate professor, Maastricht University

Dr. Anna Roukens, internist-infectiologist, LUMC

Natasja van Holten, HIV nurse specialist and president of the Netherlands Nurses & Caregivers.

Prof. Dr. Annelies Verbon, professor of infectious diseases and chairman of the Dutch Association of internist-infectiologists

SECTION 1: BACKGROUND INFORMATION

First we will ask about your background.

1. In which country do you work?
2. In which city do you work?
3. How old are you? … years
4. What is your gender?

☐ Woman ☐ Man ☐ Other

1. a. What is your current job? …

b. What department are you working in? …

c. In which hospital are you working?

d. In which country are you working? …

e. In which city are you working? …

1. How many years have you worked in healthcare? … years
2. Have you ever worked in a clinic/hospital/department that specialized in HIV care and treatment?

☐ Yes ☐ No

1. If low prevalence, use question a, If high prevalence, use question b
   1. In the past 12 months, approximately how many HIV-positive patients did you provide with care or services? …
   2. In a typical week, approximately how many HIV-positive patients do you provide with care or services? …
2. Did you ever receive training in the following subjects? (Check all that apply.)
   1. HIV stigma and discrimination ☐
   2. Infection control and universal precautions ☐

(including post-exposure prophylaxis)

- 1. Patients’ informed consent, privacy, and confidentiality ☐
  2. Key population stigma and discrimination ☐

SECTION 2: INFECTION CONTROL

Now we will ask you about infection concerns in your health facility.

1. How worried would you be about getting HIV if you did the following?

*If any of the following is not one of your job responsibilities, please select “Not applicable.”*

- 1. Touched the clothing of a patient living with HIV

☐ Not worried ☐ A little worried ☐ Worried ☐ Very worried ☐ Not applicable

- 1. Dressed the wounds of a patient living with HIV

☐ Not worried ☐ A little worried ☐ Worried ☐ Very worried ☐ Not applicable

- 1. Drew blood from a patient living with HIV

☐ Not worried ☐ A little worried ☐ Worried ☐ Very worried ☐ Not applicable

1. Do you typically use any of the following measures when providing care or services for a patient living with HIV?
   1. Avoid physical contact

☐ Yes ☐ No ☐ Not applicable

- 1. Wear double gloves

☐ Yes ☐ No ☐ Not applicable

SECTION 3: HEALTH FACILITY ENVIRONMENT

Now we will ask about practices in your health facility and your experiences working in a

facility that provides care to people living with HIV.

1. In the past 12 months have you seen a person living with HIV in your health facility?

☐ Yes go to question 13

☐ No skip to question 14

☐ Don’t know skip to question 14

1. In the past 12 months, how often have you observed the following in your health facility?
   1. Healthcare workers unwilling to care for a patient living with or thought to be living with HIV

☐ Never ☐ Once or twice ☐ Several times ☐ Most of the time

- 1. Healthcare workers providing poorer quality of care to a patient living with or thought to be living with HIV, relative to other patients

☐ Never ☐ Once or twice ☐ Several times ☐ Most of the time

SECTION 4: HEALTH FACILITY POLICIES

Now we are going to ask about the institutional policy and work environment in your facility.

1. I will get in trouble at work if I discriminate against patients living with HIV.

☐ Yes ☐ No ☐ Don’t Know

1. Do you strongly agree, agree, disagree, or strongly disagree with the following statements?
   1. There are adequate supplies in my health facility that reduce my risk of becoming infected with HIV.

☐ Strongly Agree ☐ Agree ☐ Disagree ☐ Strongly Disagree

- 1. There are standardized procedures/protocols in my health facility that reduce my risk of becoming infected with HIV.

☐ Strongly Agree ☐ Agree ☐ Disagree ☐ Strongly Disagree

1. My health facility has written guidelines to protect patients living with HIV from discrimination.

☐ Yes ☐ No ☐ Don’t Know

SECTION 5: OPINIONS ABOUT PEOPLE LIVING WITH HIV

Now we are going to ask about opinions related to people living with HIV.

1. Do you strongly agree, agree, disagree, or strongly disagree with the following statements?
   1. Most people living with HIV do not care if they infect other people.

☐ Strongly Agree ☐ Agree ☐ Disagree ☐ Strongly Disagree

- 1. People living with HIV should feel ashamed of themselves.

☐ Strongly Agree ☐ Agree ☐ Disagree ☐ Strongly Disagree

- 1. People get infected with HIV because they engage in irresponsible behaviors.

☐ Strongly Agree ☐ Agree ☐ Disagree ☐ Strongly Disagree

- 1. Women living with HIV should be allowed to have babies if they wish.

☐ Strongly Agree ☐ Agree ☐ Disagree ☐ Strongly Disagree

SECTION 6: DISCUSSING HIV WITH PATIENTS

Now we are going to ask about level of difficulty related to discussing HIV with patients.

*If any of the following is not one of your job responsibilities, please select “Not applicable.”*

1. How difficult do you find the following topics to discuss with your patients?
2. The possibility of an HIV infection

☐ Very Easy ☐ Easy ☐ Difficult ☐ Very Difficult ☐ Not applicable

1. The need to test for HIV

☐ Very Easy ☐ Easy ☐ Difficult ☐ Very Difficult ☐ Not applicable

1. Risk factors for HIV

☐ Very Easy ☐ Easy ☐ Difficult ☐ Very Difficult ☐ Not applicable

1. HIV-related topics when a patient is accompanied by a family member

☐ Very Easy ☐ Easy ☐ Difficult ☐ Very Difficult ☐ Not applicable
