# Supplementary material for: Unmasking Individual and Institutional HIV Stigma in Hospitals: Perspectives of Dutch Healthcare Providers
Source: AIDS Behav. 2024 Jun 13;28(9):3184–95. doi: 10.1007/s10461-024-04404-0 (PMC11390866; doi:10.1007/s10461-024-04404-0)
Supplement: Supplementary file 2 — Supplementary Material 2 [file 10461_2024_4404_MOESM2_ESM.docx]

**Full title: Unmasking individual and institutional HIV stigma in hospitals: Perspectives of Dutch healthcare providers**

**Short title: HIV stigma among Dutch healthcare providers**

C.C.E. Jordans^1^, K.J. Vliegenthart-Jongbloed^2^, A.W. van Bruggen^3^, N. van Holten^4^, J.E.A. van Beek, M. Vriesde, D. van der Sluis^4^, A. Verbon^1,5^, A.H.E. Roukens^4^, S.E. Stutterheim^6^, C. Rokx^1,2^

On behalf of the #aware.hiv study group

^1^ Department of Medical Microbiology and Infectious Diseases, Erasmus University Medical Center, 3015 CN, Rotterdam, the Netherlands
^2^ Department of Internal Medicine, Section Infectious Diseases, Erasmus University Medical Center, 3015 CN, Rotterdam, the Netherlands
^3^ Master student Infectious Diseases, Erasmus University Medical Center, Rotterdam, the Netherlands
^4^ Department of Infectious Diseases, Leiden University Medical Center, Leiden, the Netherlands

^5^ Department of Infectious Diseases, University Medical Center Utrecht, Utrecht, the Netherlands
^6^ Department of Health Promotion & Care and Public Health Institute, Maastricht University, PO Box 616, 6200 MD Maastricht, the Netherlands

**Corresponding author:** Dr. C. Rokx, c.rokx@erasmusmc.nl P.O. Box 2040, 3015 CN Rotterdam, the Netherlands, internal postal address Na901K

# Appendix B – Standard operating procedure distribution of the questionnaire

The objective of this survey research is to provide insight into HIV-related stigma among healthcare providers in hospitals. You and your colleagues can contribute to this insight. To get a broad perspective, the inclusion of as many healthcare providers of different professions (physicians, nurses, physician assistants, midwives, etc.) and of different departments is necessary.

There are different methods to distribute the questionnaire. Beneath we explain the steps that will be followed in distributing the questionnaire in Dutch hospitals. We strongly advise you to follow the same approach. However, the setting of your hospital can be different. Therefore, you can modify or add methods according to your experience and expertise to make sure the research approach fits your hospital. Please register the methods you are using to ensure transparency and to be aware of possible bias during the data collection process. When distributing the questionnaire, regardless of the method you use, be aware of the following:

- **Approach healthcare providers** with the aim of this survey to get insight into how healthcare providers feel about working with HIV patients. **You want to avoid words such as ‘stigma’ and ‘discrimination’** as these words can cause extra attention and might lead to socially desirable answers as people do not want to be labeled as actors of stigma. For example, use the following sentences:
  - “We want to get insight into healthcare providers’ approaches to managing HIV within hospital settings.”
  - “We want to know how healthcare providers feel about working with people living with HIV.”

Follow the following steps to distribute the questionnaire:

- Start with sending a mass mail with the link to the questionnaire to the head of the departments with a request to send it to all healthcare providers in the department. Include at least all departments that are included in the list of HIV indicator conditions of the European Centre for Disease Prevention And Control (See [HIV indicator conditions ECDC](https://webgate.ec.europa.eu/chafea_pdb/assets/files/pdb/20114202/20114202_d7_en_ps.pdf): https://webgate.ec.europa.eu/chafea_pdb/assets/files/pdb/20114202/20114202_d7_en_ps.pdf).
- Ask the head of the department if he can bring the questionnaire to the attention during report meetings in the morning.
- Place the link to the questionnaire on the online portal of the healthcare providers.
- Find a physician and/or nurse per department that can work as ambassadors for the #aware.hiv project. They can bring the survey to the attention during meetings. You can give them feedback on the response rate.
- If the response rate is low, request ambassadors to repeat bringing the questionnaire to colleagues’ attention. Request them to bring the questionnaire to the attention again during the report meetings or other (multidisciplinary) meetings, preferably once in the morning with a reminder by the end of the day.
- With low response rates: repeat bringing the questionnaire to the attention at least once and a maximum of three times, with an interval of at least two weeks.
- Reach out to managers, contact persons among nurses/head nurses and heads of departments, and ask them if a member of the project team can do on-site visits at the department to bring the survey to the attention during report meetings.
- After your on-site visit, ask if you can leave a folder/poster with the QR code in the working spaces of healthcare providers.
- During on-site visits ask healthcare providers to find at least one colleague to complete the questionnaire as well.

Lastly, be aware of the fact that healthcare providers who are specialized in HIV care might be more willing to contribute to the research project. Check multiple times during the data collection process which healthcare provider groups have responded and which have not. Use this feedback to reach out to missing participant groups. This will contribute to representative groups of each profession, department, or age group.
