# Supplementary material for: Unmasking Individual and Institutional HIV Stigma in Hospitals: Perspectives of Dutch Healthcare Providers
Source: AIDS Behav. 2024 Jun 13;28(9):3184–95. doi: 10.1007/s10461-024-04404-0 (PMC11390866; doi:10.1007/s10461-024-04404-0)
Supplement: Supplementary file 3 — Supplementary Material 3 [file 10461_2024_4404_MOESM3_ESM.docx]

**Full title: Unmasking individual and institutional HIV stigma in hospitals: Perspectives of Dutch healthcare providers**

**Short title: HIV stigma among Dutch healthcare providers**

C.C.E. Jordans^1^, K.J. Vliegenthart-Jongbloed^2^, A.W. van Bruggen^3^, N. van Holten^4^, J.E.A. van Beek, M. Vriesde, D. van der Sluis^4^, A. Verbon^1,5^, A.H.E. Roukens^4^, S.E. Stutterheim^6^, C. Rokx^1,2^

On behalf of the #aware.hiv study group

^1^ Department of Medical Microbiology and Infectious Diseases, Erasmus University Medical Center, 3015 CN, Rotterdam, the Netherlands
^2^ Department of Internal Medicine, Section Infectious Diseases, Erasmus University Medical Center, 3015 CN, Rotterdam, the Netherlands
^3^ Master student Infectious Diseases, Erasmus University Medical Center, Rotterdam, the Netherlands
^4^ Department of Infectious Diseases, Leiden University Medical Center, Leiden, the Netherlands

^5^ Department of Infectious Diseases, University Medical Center Utrecht, Utrecht, the Netherlands
^6^ Department of Health Promotion & Care and Public Health Institute, Maastricht University, PO Box 616, 6200 MD Maastricht, the Netherlands

**Corresponding author:** Dr. C. Rokx, c.rokx@erasmusmc.nl P.O. Box 2040, 3015 CN Rotterdam, the Netherlands, internal postal address Na901K

**Appendix**

Appendix A: Questionnaire HIV stigma – see word document questionnaire English

Appendix B: Standard operating procedure

**Supplementary data**

**Supplementary table 1: baseline characteristics of participants that did not complete any of the stigma questions**

|  | **Participants** |
| --- | --- |
| **Age, years**  Median (IQR) | 33 (25 - 43) |
| **Age groups, years, n (%)**  ≤ 25  26 – 45  ≥ 46 | 4 (23.5)  10 (58.8)  3 (17.6) |
| **Gender, n (%)**  Female Male | 14 (82.4)  3 (17.6) |
| **Occupation, n (%)**  Nurse  Resident  Medical specialist Other | 6 (35.3)  3 (17.6)  1 (5.9)  7 (41.2) |
| **Department(s) , n (%)**  Internal medicine  Emergency department  Cardiothoracic surgery  Pulmonology  Otorhinolaryngology  Oncology  Surgery  Gastroenterology/hepatology  Neurosurgery  Infectious diseases  Intensive care  Short stay  Dermatology  Other* | -  -  -  2 (11.8)  3 (17.6)  -  2 (11.8)  1 (5.9)  -  3 (17.6)  -  -  -  6 (35.3) |
| **Worked in more than one department, n (%)**  Yes | 2 (11.8) |
| **Time worked in healthcare, years**  Median (IQR) | 6 (2 – 18) |
| **Time worked in healthcare, years grouped, n (%)**  < 5  5 - 10  > 10  Missing | 8 (47.1)  1 (5.9)  7 (41.2)  1 (5.9) |
| **Number of treated patients with HIV per year**  Median (IQR) | 3 (1 – 10) |
| **City of work, n (%)**  Rotterdam  Leiden | 14 (82.4)  3 (17.6) |
| **Received training on the following topics: , n (%)**  HIV stigma and discrimination  Infection control and universal precautions  Patient’s informed consent, privacy, and confidentiality  Key population stigma and discrimination  **Received any of above training** | 2 (11.8)  4 (23.5)  1 (5.9)  0 (0.0)  5 (29.4) |

* Other included Cardiology, Endocrinology, Geriatrics, Gynaecology, Hematology, Medical microbiology, Nephrology, Neurology, Ophthalmology, Pediatrics, Psychiatrics, and other
HIV = human immunodeficiency virus

**Supplementary table 2: Prevalence of HIV stigma indicators including all answer options**

| ***The six HIV stigma indicators measured by an adapted version of the standardized questionnaire ‘Measuring HIV stigma and discrimination among health facility staff: indicator monitoring tool’ **** | | | | | | |
| --- | --- | --- | --- | --- | --- | --- |
| **Immediately actionable causes** | | | | | | |
| ***Fear of HIV infection*** | | | | | | |
| **Leven of worry when conducting the following activities:** | **Not worried,**  **n (%)** | **A little bit worried,**  **n (%)** | **Worried,**  **n (%)** | **Very worried, n (%)** | **Not applicable,**  **n **** | |
| Touch clothing of a patient living with HIV | 350 (90.4) | 32 (8.3) | 4 (1.0) | 1 (0.3) | 1 | |
| Dress wounds of a patient living with HIV | 127 (34.6) | 154 (42.0) | 55 (15.0) | 31 (8.4) | 1 | |
| Draw blood from a patient living with HIV | 101 (27.5) | 162 (44.0) | 59 (16.0) | 46 (12.5) | 1 | |
| ***Institutional-level facilitators and barriers (facility policy)*** | | | | | | |
| **Level of agreement with the following statements:** | **Strongly agree,**  **n (%)** | **Agree,**  **n (%)** | **Disagree,**  **n (%)** | **Strongly disagree,**  **n (%)** | **Missing,**  **n (%)** | |
| There are adequate supplies in my health facility that reduce my risk of becoming infected with HIV | 191 (49.2) | 170 (43.8) | 16 (4.1) | 2 (0.6) | 9 (2.3) | |
| There are standardized procedures/protocol in my health facility that reduce my risk of becoming infected with HIV | 151 (38.9) | 202 (52.1) | 23 (5.9) | 3 (0.8) | 9 (2.3) | |
| **Health facility’s policies on discrimination** | **Yes,**  **n (%)** | **No,**  **n (%)** | **Don’t know, n (%)** | **Missing,**  **n (%)** | | |
| I will get in trouble at work if I discriminate against patients living with HIV | 217 (55.9) | 16 (4.1) | 145 (37.4) | 10 (2.6) | | |
| My health facility has written guidelines to protect patients living with HIV from discrimination | 68 (17.5) | 14 (3.6) | 297 (76.6) | 9 (2.3) | | |
| ***Attitudes (stereotype and prejudice)*** | | | | | | |
| **Level of agreement with the following statements:** | **Strongly agree,**  **n (%)** | **Agree,**  **n (%)** | **Disagree,**  **n (%)** | **Strongly disagree,**  **n (%)** | **Missing,**  **n (%)** | |
| Most people living with HIV do not care if they infect other people | 2 (0.5) | 25 (6.5) | 231 (59.5) | 113 (29.1) | 17 (4.4) | |
| People living with HIV should feel ashamed of themselves | 0 (0) | 3 (0.8) | 68 (17.8) | 299 (77.1) | 17 (4.4) | |
| People get infected with HIV because they engage in irresponsible behaviors | 1 (0.3) | 37 (9.5) | 184 (47.5) | 148 (38.1) | 18 (4.6) | |
| Women living with HIV should be allowed to have babies if they wish | 148 (38.0) | 181 (46.6) | 35 (9.0) | 6 (1.5) | 18 (4.6) | |
| **Manifestations (discrimination)** | | | | | | |
| ***Self-reported use of unnecessary infection control measures*** | | | | | | |
| **Typically use any of the following measures when providing services to patients living with HIV:** | **Yes,**  **n (%)** | **No,**  **n (%)** | | | **Not applicable, n **** | |
| Avoid physical contact | 24 (6.5) | 347 (93.5) | | | 17 | |
| Wear double gloves | 57 (15.4) | 312 (84.6) | | | 19 | |
| ***Observed discrimination*** | | | | | | |
| **In <12 months have you seen a person living with HIV** | **Yes,**  **n (%)** | **No,**  **n (%)** | | | **Don’t know,**  **n (%)** | |
| Have you seen a person living with HIV in your health facility | 303 (78.1) | 56 (14.4) | | | 29 (7.5) | |
| **In <12 months how often observed the following at your health facility** | **Never,**  **n (%)** | **Once or twice,**  **n (%)** | **Several times,**  **n (%)** | **Most of the time,**  **n (%)** | **Missing,**  **n (%)** | |
| Healthcare providers unwilling to care for a patient living with HIV | 290 (74.4) | 10 (2.6) | 2 (0.5) | 0 (0) | 86 (22.2) | |
| Healthcare providers providing poorer quality of care to a patient living with HIV compared to other patients | 279 )71.9) | 20 (5.2) | 4 (1.0) | 0 (0) | 85 (21.9) | |
| **Experienced barriers** | | | | | | |
| ***Discussing HIV and risk factors with patients*** | | | | | | |
| **Level of difficulty for healthcare providers discussing the following topics:** | **Very easy, n (%)** | **Easy,**  **n (%)** | **Difficult,**  **n (%)** | **Very difficult,**  **n (%)** | **Missing, n (%)** | **Not applicable, n **** |
| The possibility of an HIV infection | 35 (11.2) | 133 (42.5) | 111 (35.5) | 11 (3.5) | 23 (7.3) | 75 |
| The need to test for HIV | 73 (22.9) | 170 (53.3) | 51 (16.0) | 2 (0.6) | 23 (7.2) | 69 |
| Risk factors for HIV | 55 (17.1) | 153 (47.7) | 83 (25.8) | 7 (2.2) | 23 (7.2) | 67 |
| HIV-related topics when a patient is accompanied by a family member | 17 (5.2) | 79 (24.4) | 171 (52.8) | 34 (10.5) | 23 (7.1) | 64 |

* Reference: <https://www.healthpolicyproject.com/pubs/49_StandardizedBriefQuestionnaireMeasuringSD.pdf>
** Not applicable will be excluded from the denominator, as the response to this question does not apply to the healthcare provider answering this question
HIV = Human immunodeficiency virus
